# VISTA H-Score Is Significantly Associated with a 5-Year DFS Rate in Oral Squamous Cell Carcinoma

**DOI:** 10.3390/jcm12041619

**Published:** 2023-02-17

**Authors:** Anna Starzyńska, Bartosz Kamil Sobocki, Monika Sakowicz-Burkiewicz, Barbara Alicja Jereczek-Fossa, Daniela Alterio, Olga Szot, Aleksandra Korwat, Rafał Pęksa

**Affiliations:** 1 Department of Oral Surgery, Medical University of Gdansk, 7 Dębinki Street, 80-211 Gdansk, Poland; 2 Department of Molecular Medicine, Medical University of Gdansk, 7 Dębinki Street, 80-211 Gdansk, Poland; 3 Division of Radiotherapy, IEO European Institute of Oncology, IRCCS, 435 Ripamonti Street, 20-141 Milan, Italy; 4 Department of Oncology and Hemato-Oncology, University of Milan, 7 Festa del Perdono Street, 20-112 Milan, Italy; 5 Department of Pathology, Medical University of Gdansk, 17 Smoluchowskiego Street, 80-214 Gdansk, Poland

**Keywords:** VISTA, oral squamous cell carcinoma, prognosis, biomarkers

## Abstract

Oral squamous cell carcinoma (OSCC) is the most common type of oral cancer in the world. Despite its prevalence, it is often recognized in advanced stages (III or IV) when it has already spread to local lymph nodes. In this study, we investigate the V-domain Ig suppressor of T cell activation (VISTA) as a potential prognostic factor in OSCC. Tissue samples were collected from 71 oral squamous cell carcinoma patients to determine protein expression levels (using immunochemistry and the semi-quantitative H-score method). Moreover, RT-qPCR was additionally performed in 35 patients. Clinical factors in our cohort study had no impact on VISTA expression. However, VISTA expression is largely correlated with Il-33 levels in tumor cells and lymphocytes and with PD-L1 in tumor cells. The impact of VISTA expression on overall survival (OS) is rather limited, but in the case of a 5-year survival rate, a significant association has been proven. VISTA seems to be a rather weak clinicopathological marker but needs further evaluation in the context of survival. In addition, the potential of VISTA combination with Il-33 or PD-L1 should be further investigated in OSCC.

## 1. Introduction

**Figure 1:**
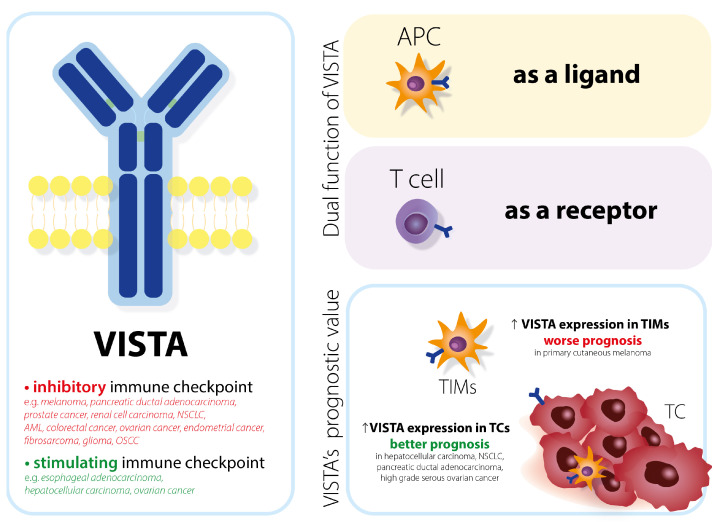
Main functions of VISTA. Depending on the cell type, VISTA can act as a ligand and as a receptor. VISTA as an immune checkpoint differs among cancers. Whether it has an inhibitory or stimulating function determines the progression of carcinogenesis. In certain types of cancer, VISTA expression makes the prognosis better. VISTA = V-domain Ig suppressor of T cell activation; APC = antigen presenting cell.

V-domain Ig suppressor of T cell activation (VISTA) is a novel immune checkpoint target in onco-immunotherapy. It is a homolog of both programmed death 1 (PD-1) and programmed death ligand 1 (PD-L1) [1] and it belongs to the B7 family, even though it does not have an immunoreceptor tyrosine-based activation/inhibitory motif [2]. VISTA consists of an extracellular part, Ig-V domain and stalk region, transmembrane segment, and cytoplasmic domain (with potential sites for protein kinase C and casein kinase 2 phosphorylation sites [3]). The structure of the immunoglobulin explains its ability to act as a receptor on T cells and as a ligand on antigen-presenting cells [4] (Figure 1).

VISTA is mainly expressed in hematopoietic cells. The highest VISTA expression among lymphocytes T is observed in naive CD4+ and Foxp3+ regulatory T cells. VISTA protein can be present in tumor-infiltrating macrophages (TIMs) or tumor cells (TCs) [5]. The localization of heightened VISTA protein expression correlates with overall survival (OS). VISTA expression in TCs in hepatocellular carcinoma [1], non-small cell lung cancer [6], pancreatic ductal adenocarcinoma with favorable survival [5], and high-grade serous ovarian cancer [7] was associated with longer OS. However, VISTA expression in TIMs in primary cutaneous melanoma resulted in a worse prognosis [8].

VISTA expression and VISTA protein have been evaluated in many cancers. However, their function differs among them. VISTA acts as an inhibitory immune checkpoint by suppressing T cells and enabling cancer’s immune escape in melanoma [9], pancreatic ductal adenocarcinoma [10], prostate cancer [11], renal cell carcinoma [12], non-small cell lung cancer [6], acute myeloid leukemia [13], colorectal cancer [4], ovarian cancer, endometrial cancer [14], fibrosarcoma [15], glioma [16], and oral squamous cell carcinoma [17].

Despite being a homolog of PD-L1 (another inhibitory immune checkpoint), VISTA does not overlap with the PD-1 regulatory pathway [18]. After blocking the PD-1 pathway in prostate cancer patients, there was an increase in the number of VISTA+ lymphocytes, which resulted in acquiring resistance to immune checkpoint blockade [9,11]. Anti-VISTA antibodies can be applied not only in mono-therapy, but also in poly-therapy [19], the case of resistance to anti-PD-1 and anti-CLTA4 treatment, and complementary therapy [1,14].

VISTA is a stimulating immune checkpoint and evokes an immune response to cancerous tissues in cancers such as esophageal adenocarcinoma [20], hepatocellular carcinoma [7], and ovarian cancer [1].

Apart from being a potential prognostic and therapeutic target in cancer treatment, VISTA’s immune-suppressing properties might also have therapeutic potential in treating autoimmune diseases and preventing acute graft-versus-host disease [3].

## 2. Materials and Methods

### 2.1. Characteristics of the Study Group

**Figure 2:**
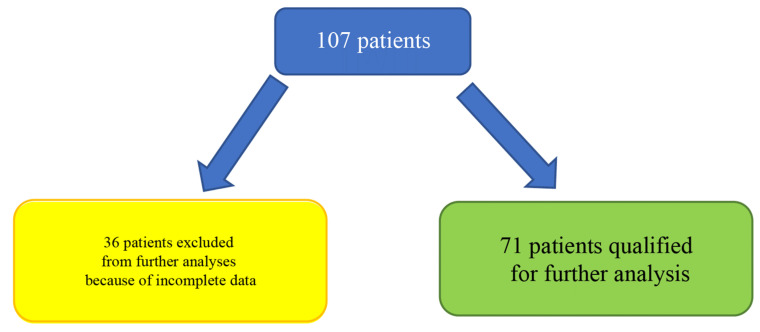
Flow chart describing qualification of patients to all parts of the analysis.

This study was accepted and approved by the local ethics committee of the Medical University of Gdańsk, Poland [NKBBN/59-747/2021]. The 36 patients were excluded from the analysis due to the incomplete clinical data (about TNM, grade, or co-morbidities) and finally, the analysis was retrospectively made in the group of 71 Caucasian patients. The qualification of the patients is depicted in Figure 2. In this patient group (71 patients), immunohistochemistry (IHC) staining was performed, whereas RT-qPCR in real-time was additionally made in 35 patients. Due to the limited number of biological materials, it was possible to perform RT-qPCR only for 35 patients from the whole investigated population. The material was collected during surgical resections or diagnostic biopsy procedures. These patients were hospitalized at the Maxillofacial Surgery Department at the University Clinical Centre in Gdańsk from 2007 to 2012. The information about co-morbidities and necessary clinical data for OSCC were collected in order to provide sufficient and precise information about the investigated group and to assess the impact of that factors on VISTA expression. TNM stages were evaluated according to the 8th edition of the AJCC Cancer Staging Manual. In addition, the material was graded (G1–G4) by well-qualified pathologists with relevant clinical experience.

### 2.2. Immunohistochemistry

All of the tissue probes were fixed in 4% buffered formalin and embedded in low-melting paraffin. Tissue microarrays were constructed with a Manual Tissue Arrayer MTA-1 device (Beecher Instruments Inc., Sun Prairie, WI, USA) with 1.5 mm core needles. The Leica SM 200 microtome was used to cut the material into sections of 4 μm thickness. Representative tumor areas were selected and stained with antibodies against VISTA (clone D5L5T, 1:300 dilution, Cell Signaling). The staining was conducted with applicable positive and negative control. During the procedure of staining, the Dako EnVision Flex/HRP system was used. Before the staining, the following procedures such as incubation (24 h, 37 °C), deparaffinization, rehydration, the antigen retrieval heat-induced epitope retrieval method (PTLink, Dako), and blocking endogenous peroxidase (3% H_2_O_2_, 5 min) were performed. The adequate positive controls (histologically normal tonsil) were incorporated in TMAs without primary antibodies that were used as a negative control.

### 2.3. H-Score Analysis

The microscope glass slides were analyzed under the light microscope and the level of VISTA on lymphocytes was evaluated. The representative tumor areas were the ones that contained cancer and stroma components. For each core, the intensity (weak, medium, strong) and percentage of the positively stained cells were quantified. The semi-quantitative H-score coefficient was calculated using the following formula: *percentage of weakly stained cells + percentage of moderately stained cells x2 + percentage of strongly stained cells ×3* [21]. The final points range from 0 to 300. The H-score review was performed independently by two pathologists. If any differences between pathologists were revealed, the third specialist would review the score.

### 2.4. Evaluation of mRNA Expression

The RNA was isolated from 35 freshly cut FFPE samples (into 8 to 10,5 µm thick fragments) with the use of RNeasy FFPE Mini Kit by QIAGEN N (Qiagen GmbH, Hilden, Germany) according to the manual user. Then, the amount of total RNA was fluorometrically detected with a Quant-iT kit (Thermo Fisher Scientific, Warszawa, Poland) according to the protocol and manual user. The gene expression level of *VISTA* was determined by RT-qPCR in real-time performed in a Light Cycler 480 II (Roche Diagnostics International Ltd., Rotkreutz, Switzerland) using Path-IDTM Multiplex One-Step RT-PCR Kit (Thermo Fisher Scientific, Warszawa, Poland) and Universal ProbeLibrary for Human (probe #61) (Roche Diagnostics GmbH, Mannheim, Germany), and gene-specific intron-spanning primers ((F) ATCCCTGCTCTTCGCTCT, (R) CCTCGGGACAGACATACAGG). VISTA expression was normalized with the reference gene *ACTB*, using the Universal ProbeLibrary Human *ACTB* Gene Assay (Roche Diagnostics GmbH, Mannheim, Germany). The reverse transcription program was 48 °C—10 min and 95 °C—10 min. The amplification program was 95 °C—15 s and 60 °C—45 s for 50 cycles. Data were processed with Light Cycler 480 II software 2.0.

### 2.5. Statistical Analysis

All of the statistical analyses were performed with STATISTICA 13.3 (StatSoft Inc., Tulsa, OK, USA), except for the correlation and survival analyses made in SPSS 28.0.0.0 (190, IBM, Armonk, NY, USA). In this study *p* < 0.05 was found as statistically significant. The W Shapiro–Wilk test was used for verification of the normal distribution of data. The impact of different clinical factors was assessed with the Mann–Whitney U or Student’s *t*-test test and Kruskal–Wallis ANOVA or one-way ANOVA tests when applicable. The correlation analysis was performed on the basis of Spearman’s rank correlation. In survival analysis, the Kaplan–Meier Curve with the log-rank test, univariate, and multiple Cox regression (for available clinical factors) models were applied. The chi-square test of independence was used to compare 5-year disease-free survival between the groups.

## 3. Results

### 3.1. Clinical Characteristics of Patients and Associations between VISTA Expression and Clinical Factors

Firstly, we wanted to investigate any possible associations between *VISTA* mRNA expression or VISTA H-score and clinical factors. Our analysis indicated that none of the clinical factors has a statistically significant impact on VISTA H-score and *VISTA* mRNA expression. Only the presence of surgical resection and diabetes was close to being significant in the case of VISTA H-score (respectively *p* = 0.07 and *p* = 0.08). Whereas smoking cigarettes was close to being relevant for mRNA expression (*p* = 0.064). In order to assess VISTA H-score and *VISTA* mRNA expression as the potential clinicopathological markers, we compared VISTA H-score in reference to Grade, Stage, pT, and pN. However, the lack of significant impact of any parameter was observed.

**Figure 3:**
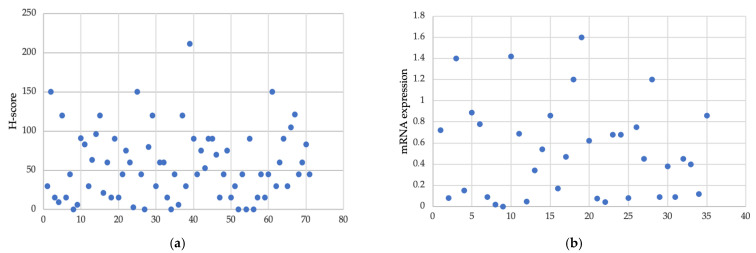
Distribution of VISTA H-score and *VISTA* mRNA expression in the group. (**a**) H-score level in the group of 71 patients. (**b**) mRNA expression in the group of 35 patients.

The median of the VISTA H-score in the cohort was 45 (0–211), whereas the mean of *VISTA* mRNA expression was 0.53 (0.001–1.6). The lack of protein expression was noted in six cases. The histogram of the VISTA H-score and *VISTA* mRNA expression level are shown in Figure 3.

The detailed characteristics of cohorts included in this study are depicted in Table 1 and Table 2.

### 3.2. VISTA Expression Is Weakly Correlated with PD-L1 and Il-33 Expression

**Figure 4:**
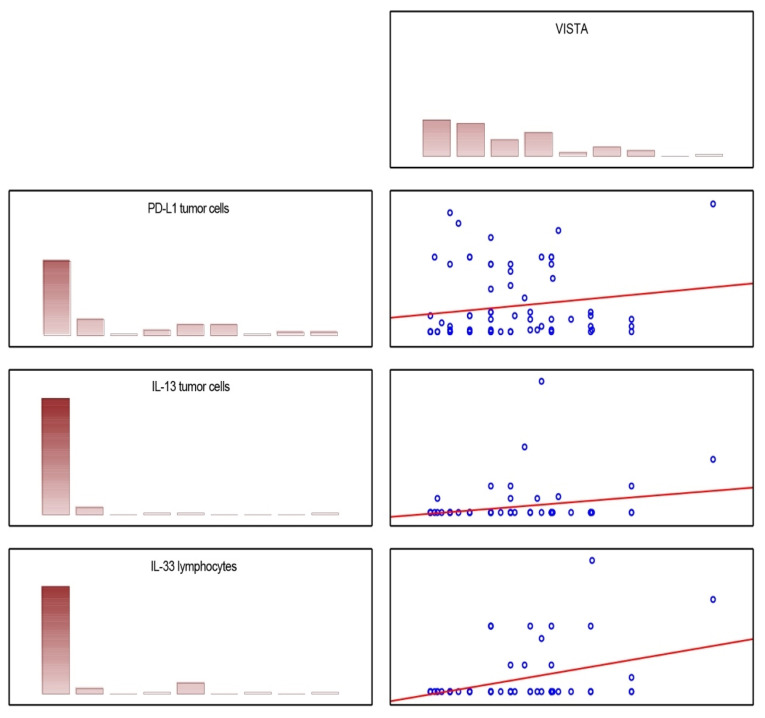
Scatter plot matrix describing significant correlations of VISTA with molecules analyzed in our previous studies in the same cohort.

Having the results of VISTA H-score and mRNA, we tried to correlate them with the results from our previous studies regarding ZNF-281 [21], PD-L1, and Il-33 [22] based on the same cohort of patients. The Spearman correlation matrix showed that significant but weak correlations between VISTA H-score and PD-L1 H-score (tumor cells, *p* = 0.027; R = 0.263) or Il-33 H-score (tumor cells *p* = 0.04; R = 0.25) were found. A moderate correlation was observed between VISTA H-score and Il-33 H-score levels (lymphocyte, *p* = 0.002; R = 0.37). These correlations are depicted in Figure 4.

### 3.3. Survival Analysis

**Figure 5:**
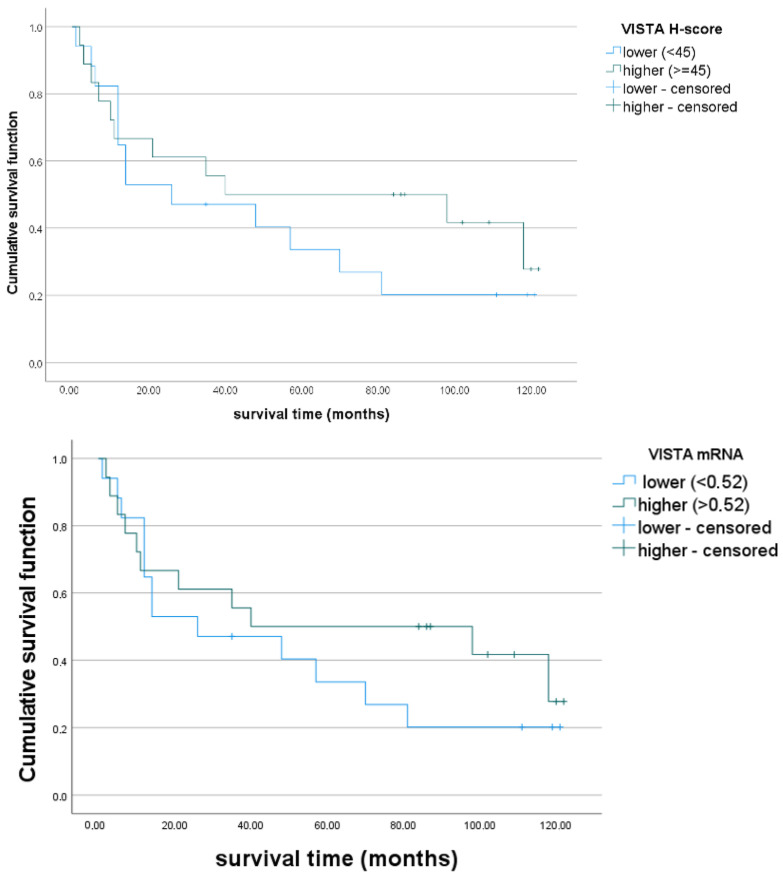
Overall survival in the two groups with higher and lower *VISTA* expression: Kaplan– Meier curves. Patients were divided according to the median of the H-score distribution into two groups with higher (H-score > 45, *n* = 33) and lower (H-score ≤ 45, *n* = 38) VISTA expression. The log-rank test showed no significant difference between groups. Regarding mRNA expression, we divided the cohort into two groups with higher (>0.52, *n* = 16) and lower mRNA (<0.52, *n* = 19) expression. Similarly, the difference between groups was non-significant in the log-rank test.

The number of deaths, in the group classified in this analysis, was 52 (73.2%). The median of the OS was 40 (1–135) months, and 5-year disease free-survival (DFS) was 42.25%. Taking into consideration the H-score, we divided our patients’ population into two groups according to VISTA expression: higher (H-score > 45, *n* = 33) and lower (H-score ≤ 45, *n* = 38). Evaluating *VISTA* mRNA, we divided the cohort into two groups with higher (>0.52, *n* = 16) and lower mRNA (<0.52, *n* = 19) expression. However, the Kaplan–Meier Curve and the log-rank test showed that the impact of VISTA H-score level on OS was non-significant (log-rank, *p* = 0.48). Similarly, the impact of *VISTA* mRNA on OS was non-significant (*p* = 0.396). Kaplan–Meier curves for the two groups are depicted in Figure 5.

The univariate cox regression model confirmed that both VISTA H-score and *VISTA* mRNA impact on OS were limited in our group (respectively, *p* = 0.133; *p* = 0.073). We also adjusted VISTA expression in the multivariate Cox regression model for stage, grade, age, sex, surgery status, diabetes, and hypertension, obtaining a lack of significance. In our group, the parameters as stage and surgery status were the most relevant ones influencing OS. The results are depicted in Table 3.

Although the impact of VISTA expression on OS was limited, the 5-year DFS in the group of patients with lower VISTA expression was 32.4 %, whereas in the group of patients with higher VISTA expression, it was 52.9 %, and there was a significant difference between the groups (*p* = 0.05). Although we observed a similar tendency, we did not confirm a significant difference between the groups in the case of *VISTA* mRNA expression (*p* = 0.62, higher (56.3%) vs lower (42.1%).

## 4. Discussion

Nowadays, the articles concerning VISTA expression and precising a role of this immune checkpoint in OSCC are limited in number. In addition, there is a lack of consensus on the matter of the prognostic value of VISTA in OSCC among studies published in the literature. What is more, there are only a few publications that describe the conducted analysis of intra-tumoral expression according to clinical parameters in order to assess the role of VISTA as the clinicopathological biomarker of OSCC. In our study, we would like to provide data for further analyses of contradictions in VISTA’s research.

Wu et al. indicated that VISTA expression is significantly higher in OSCC than in normal adjacent tissue and that VISTA expression in the primary tumor was correlated with lymph node status. On the contrary, our results suggest that there is no association between VISTA expression and N classification. Moreover, the study of Wu et al. also showed that VISTA expression is not correlated with stage, grade, or T classification [17]. Our study confirmed these results.

The role of VISTA as the prognostic factor of OSCC is still undetermined. We found only two articles describing VISTA in the context of its prognostic role [17,23]. Log-rank analysis conducted by Wu et al. showed that there is no difference in OS related to VISTA expression [17]. Our analysis confirmed these results. All of the available studies about VISTA in OSCC indicate that VISTA is not an independent predictor of OS, like in our cohort. In a Wuerdemann et al. study, VISTA expression showed a significant impact on OS only in the univariate Cox regression model, whereas in a Wu et al. study, only in combination with CD8 level [17,23]. In our cohort, even in the univariate Cox regression model, we did not confirm that VISTA significantly influences OS, taking into consideration both mRNA and H-score data. However, the analysis of the 5-year DFS rate showed that patients with higher H-score had a significantly higher 5-year DFS rate. The lack of significance in the case of *VISTA* mRNA might be associated with the much smaller size of the group.

The potential clinical application of VISTA was assessed by Kondo et al. In this study, the blockage of VISTA alone was ineffective in reducing tumor growth. However, its blockage efficiently induced CD8+ T cell activation. Moreover, the combination of VISTA and CTLA-4 blockade caused tumor regression and inhibited Tregs recruitment. Taking it into consideration, future translational studies should investigate VISTA with CTLA-4 expression together [18].

The analysis of the correlation between VISTA and other markers of OSCC showed that higher levels of VISTA H-score were associated with higher levels of IL-33 expression on both tumor cells and lymphocytes and PD-L1 assessed on tumor cells. Il-33 plays an alarming signal molecule role which is engaged in the tumor-associated inflammation process. Molecules such as Il-33′s activity may be one of the major reasons for tumor immune tolerance. Wen et al. showed that accumulation of Il-33+ cells is associated with increased Treg infiltration, stimulation of suppressive cytokine production, and the enhancement of Treg-mediated suppression of proliferation in head and neck squamous cell carcinomas [24]. Moreover, Ding et al. investigated that in the co-culture system, Il-33 knockdown decreased stromal fibroblast activation and subsequently reduced tumor cell proliferation [25]. VISTA can be also treated as an immunosuppressive agent. VISTA-Fc fusion protein and overexpression of VISTA in cells were associated with limited T cell activation, proliferation, and cytokine production [26]. In addition, Kondo et al. indicated that blockage of VISTA decreased Treg level and inhibited tumor growth of melanoma cell lines [18]. PD-L1 also acts as an inhibitor of T cell activation and it is a well-known target for immunotherapeutic agents. Many studies showed that it can be treated as a potential prognostic and predictive molecule.

The widely investigated mechanism is the immunosuppressive interaction between PD-L1 on tumor cells with PD-1 on CD8+ T cells [27,28]. Until now, one inhibitor of PD-L1, PD-L2, and VISTA, known as CA-170, showed preclinical anti-tumor efficacy [29].

Interestingly, higher expression of VISTA in glioma was correlated with higher grades and worse overall survival [30]. On the other hand, in triple-negative breast cancer, higher VISTA expression was associated with prolonged relapse-free survival and overall survival times [31]. In colorectal cancer, higher VISTA expression was correlated with lower grades, early tumor stage, and prolonged survival in the investigated cohort [32]. Finally, the systematic review and meta-analysis by Xin-Lin He et al. assessed the prognostic role of VISTA in solid tumors, including ten studies and 2440 different cancer patients. The pooled results showed that high expression of VISTA was associated with favorable overall survival [33]. Our study confirmed that conclusion in terms of 5-year DFS. The differences show that VISTA’s prognostic role may vary depending on the type of cancer and should be investigated and confirmed for each cancer type separately in order to avoid confusion.

Probably, all the molecules indicated above play a role in maintaining an immunosuppressive state in the tumor microenvironment, and also in OSCC. Perhaps the co-inhibition of indicated above pathways may be therapeutically more effective than any of the molecules alone. However, further preclinical and then clinical studies are needed in order to confirm the conclusions drawn from the correlation analysis and potential synergism in the inhibition of the potential combination of these molecules.

## 5. Conclusions

The present study showed that VISTA application as a clinicopathological marker can be rather limited. Our survival analysis was consistent with other studies and showed that VISTA is not the independent predictor of hazards. The impact of VISTA on OS was not shown even in the univariate Cox regression model. However, we revealed that the association between VISTA expression level and 5-year DFS is statistically significant and that a higher VISTA H-score indicates a higher 5-year DFS rate. This result should be further explored. Moreover, in our study, we have demonstrated that VISTA H-score correlates with Il-33 and PD-L1 levels in tumor cells and Il-33 levels in lymphocytes. Further studies are needed to confirm these findings.

## Figures and Tables

**Table 1:** The impact of different factors on VISTA H-score (protein) level. Clinical characteristics of the 71-patient group with H-score.

Clinical Factor						*p*
N (%)						
**Sex**	**Female**	22 (31.0)				0.7
	**Male**	49 (69.0)				
Median (range)						
**Age**		60 (30–90)				0.99
0 = no, 1 = yes (%), NA = not available						
**Radiotherapy**		**0:** 32 (45.1)	**1:** 36 (50.7)		**NA:** 2 (2.8)	0.72
**Chemotherapy**		**0:** 62 (87.3)	**1:** 6 (8.5)		**NA:** 2 (2.8)	0.25
**Surgical resection**		**0:** 16 (22.5)	**1:** 55 (77.5)			0.07
(1–3):N(%)						
**Grade**		**1:** 32 (45.1)	**2:** 32 (45.1)		**3:** 7 (9.9)	0.51
(1–4):N(%)						
**AJCC Stage**		**1:** 17 (23.9)	**2:** 15 (21.1)	**3:** 12 (16.9)	**4:** 27 (38.0)	0.66
**T** (1–4):N(%), **N** (0–3):N(%)						
**Classification**	**T**	**1:** 12 (16.9)	**2:** 26 (36.6)	**3:** 14 (19.7)	**4:** 19 (26.8)	0.86
	**N**	**0:** 29 (40.8)	**1:** 13 (18.3)	**2:** 25 (35.2)	**3:** 4 (5.6)	0.82

For the group of patients, *p*-value was estimated to find any significant difference in VISTA expression level according to Grade, AJCC Stage, T and N classifications with the Kruskal–Wallis one-way ANOVA test, and according to other parameters with the Mann–Whitney U test.

**Table 2:** The impact of different factors on VISTA mRNA level. Clinical characteristics of the group considered 35 patients with mRNA.

Clinical Factor						*p*
N (%)						
**Sex**	**Female**	13 (37.1)				0.35
	**Male**	22 (62.7)				
Median (range)						
**Age**		60 (30–90)				0.56
0 = no, 1 = yes (%), NA = not available						
**Radiotherapy**		**0:** 18 (51.4)	**1:** 15 (42.9)		**NA:** 2 (5.7)	0.59
**Chemotherapy**		**0:** 30 (85.7)	**1:** 3 (8.6)		**NA:** 2 (5.7)	0.51
**Surgical resection**		**0:** 7 (20.0)		**1:** 28 (80.0)		0.56
(1–3):N(%)						
**Grade**		**1:** 19 (54.3)	**2:** 13 (37.1)		**3:** 3 (8.6)	0.98
(1–4):N(%)						
**AJCC Stage**		**1:** 10 (28.6)	**2:** 10 (28.6)	**3:** 4 (11.4)	**4:** 11 (31.4)	0.73
**T** (1–4):N(%), **N** (0–3):N(%)						
**Classification**	**T**	**1:** 6 (17.1)	**2:** 15 (42.9)	**3:** 8 (22.9)	**4:** 6 (17.1)	0.86
	**N**	**0:** 14 (40.0)	**1:** 4 (11.4)	**2:** 15 (42.9)	**3:** 2 (5.7)	0.86

For the group of patients, *p*-value was estimated to find any significant difference in VISTA expression level according to Grade, AJCC Stage, T and N classifications with the one-way ANOVA test, and according to other parameters with Student’s *t*-test.

**Table 3:** Univariate and multivariate Cox regression models. VISTA H-score, mRNA, and significant predictors of OS were included.

Feature	Univariate Model		Multivariate Model	
	*p*	HR (95% CI)	*p*	HR (95% CI)
VISTA H-score	0.133	0.995 (0.989–1.001)	0.083	0.993 (0.986–1.001)
VISTA mRNA	0.073	0.419 (0.162–1.084)	0.184	0.474 (0.157–1.425)
Surgical resection	0.000	0.314 (0.168–0.586)	0.037	0.428 (0.193–0.950)
stage (3 vs. 1 + 2)	0.008	2.798 (1.302–6.010)	0.008	3.336 (1.373–8.109)
cT (3 vs. 1 + 2)	0.037	2.643 (1.059–6.597)	0.359	1.788 (0.516–6.199)
cN (1 vs. 2)	0.002	1.530 (1.165–2.011)	0.148	1.303 (0.910–1.865)
pN (1 vs. 2)	0.006	1.498 (1.121–2.003)	0.131	1.576 (0.873–2.846)
Radiotherapy	0.059	1.730 (0.980–3.054)	0.316	1.399 (0.725–2.698)

## Data Availability

The data presented in this study are available on request from the corresponding author.

## Abbreviations

OSCC	oral squamous cell carcinoma
VISTA	V-domain Ig suppressor of T cell activation
PD-L1	programmed death ligand 1
PD-1	programmed death 1
APC	antigen presenting cells
TIMs	tumor infiltrating macrophages
TC	tumor cells
DFS	disease free survival
OS	overall survival
HPV	human papilloma virus
